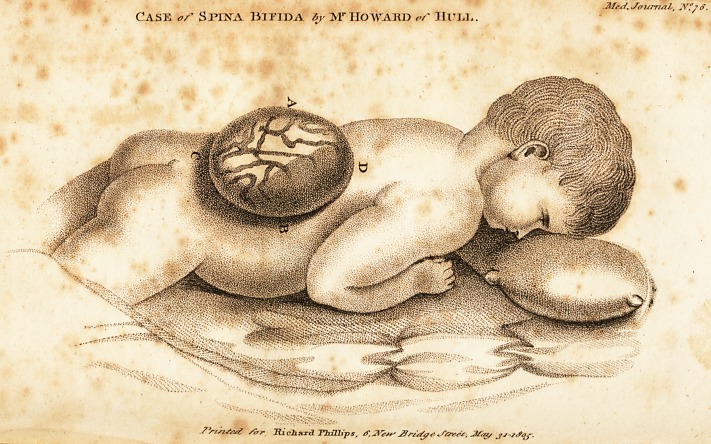# Case of Spina Bifida

**Published:** 1805-06-01

**Authors:** Joseph Howard

**Affiliations:** Member of the Royal College of Surgeons


### CASE OF SPINA BIFIDA, *communicated by Mr.* JOSEPH HOWARD, *Member of the Royal College of Surgeons.*

THE term spina bifida is generally confined to certain tumours appearing in the course of the spine. In the case in question, the tumour was situated on the lumbar vertebræ, but had not the usual characteristics, not yielding to pressure, but remaining firm. This induced me to make use of a ligature for its extirpation. After the ligature had been applied eight days, the child was seized with convulsions and died.

In order to determine whether there was a deficiency of bone, or a separation of the spinous processes of the vertebræ, I exposed such of the latter as were in the neighbourhood of the tumour. There did not, however, appear the least deficiency or separation; but a small canal or tube was found, proceeding from the vertebræ to the tumour, but not communicating with the former internally. From the length of time which the ligature had been applied, I am inclined to believe that the child's dissolution was not in consequence of the disease, or the operation; for the tumour was nearly separated, and the parts exhibited no unfavourable appearance.

### *Appearances of the Tumour, prior to the Application of the Ligature.*

It appeared to possess much irritability; neither could that transparency be observed, which is generally the case, through the tumour; it appeared very vascular, and the ramifications of blood-vessels, upon the upper portion of it, was very beautiful. It did not appear that the diameter of the tumour increased after birth. It is worthy of remark, that one side of it was of a dark ash colour, and no blood-vessels were discoverable thereon. I have sent you an exact drawing of the tumour.

---

### EXPLANATION OF THE DRAWING.

A to B the breadth of the tumour across the loins, which was 2 inches $\frac{7}{16}$; C to D the length, being 1 inch $\frac{7}{8}$; the height 2 inches $\frac{1}{16}$.

*Hull, April* 8, 1806.

*Observations*

## Figures and Tables

**Figure f1:**